# Hospital readiness for the provision of care to critically ill patients in Tanzania– an in-depth cross-sectional study

**DOI:** 10.1186/s12913-024-10616-w

**Published:** 2024-02-08

**Authors:** Karima Khalid, Carl Otto Schell, Jacquie Oliwa, Mike English, Onesmus Onyango, Jacob Mcknight, Elibariki Mkumbo, Khamis Awadh, John Maiba, Tim Baker

**Affiliations:** 1 Department of Anaesthesia, Muhimbili University of Health and Allied Sciences, Dar es Salaam, Tanzania; 2 Department of Emergency Medicine, Muhimbili University of Health and Allied Sciences, Dar es Salaam, Tanzania; 3 Ifakara Health Institute, Dar es Salaam, Tanzania; 4 Department of Global Public Health, Karolinska Institutet, Stockholm, Sweden; 5 Centre for Clinical Research Sörmland, Uppsala University, Eskilstuna, Sweden; 6 Department of Medicine, Nyköping Hospital, Nyköping, Sweden; 7 Department of Paediatrics, University of Nairobi, Nairobi, Kenya; 8 Health Services Unit, KEMRI-Wellcome Trust Research Programme, Nairobi, Kenya; 9 Department of Clinical Research, London School of Hygiene and Tropical Medicine, London, UK; 10 Health Systems Collaborative, Nuffield Department of Medicine, University of Oxford, Oxford, UK; 11 Department of Anaesthesia, Muhimbili Orthopaedic Institute, Dar es Salaam, Tanzania

**Keywords:** Critical illness, Critical care, Emergency care, Tanzania, LMICs, Global health

## Abstract

**Background:**

Critical illness is a state of ill health with vital organ dysfunction, a high risk of imminent death if care is not provided and potential for reversibility. The burden of critical illness is high, especially in low- and middle-income countries. Critical care can be provided as Essential Emergency and Critical Care (EECC)– the effective, low-cost, basic care that all critically ill patients should receive in all parts of all hospitals in the world– and advanced critical care– complex, resource-intensive care usually provided in an intensive care unit. The required resources may be available in the hospital and yet not be ready in the wards for immediate use for critically ill patients. The ward readiness of these resources, although harder to evaluate, is likely more important than their availability in the hospital. This study aimed to assess the *ward readiness* for EECC and the *hospital availability* of resources for EECC and for advanced critical care in hospitals in Tanzania.

**Methods:**

An in-depth, cross-sectional study was conducted in five purposively selected hospitals by visiting all wards to collect data on all the required 66 EECC and 161 advanced critical care resources. We defined *hospital-availability* as a resource present in the hospital and *ward-readiness* as a resource available, functioning, and present in the right place, time and amounts for critically ill patient care in the wards. Data were analyzed to calculate availability and readiness scores as proportions of the resources that were available at hospital level, and ready at ward level respectively.

**Results:**

Availability of EECC resources in hospitals was 84% and readiness in the wards was 56%. District hospitals had lower readiness scores (less than 50%) than regional and tertiary hospitals. Equipment readiness was highest (65%) while that of guidelines lowest (3%). Availability of advanced critical care resources was 31%.

**Conclusion:**

Hospitals in Tanzania lack readiness for the provision of EECC– the low-cost, life-saving care for critically ill patients. The resources for EECC were available in hospitals, but were not ready for the immediate needs of critically ill patients in the wards. To provide effective EECC to all patients, improvements are needed around the essential, low-cost resources in hospital wards that are essential for decreasing preventable deaths.

**Supplementary Information:**

The online version contains supplementary material available at 10.1186/s12913-024-10616-w.

## Introduction

Critical illness is a state of ill health with vital organ dysfunction, a high risk of imminent death if care is not provided and potential for reversibility [1]. Patients can become critically ill irrespective of their location, diagnosis, gender, or social economic status. Critical illness is estimated to affect around 30–45 [2] million adults annually– mostly in low- and middle-income countries (LMICs) [3, 4]. In the COVID-19 pandemic, the burden increased and critical illness rose up the global health agenda [5]. The mortality rate of critical illness is high– ranging between 13–85% [5–10].

Patients who are critically ill require critical care– care that supports vital organs and keeps the patient alive while definitive treatment of the underlying conditions is provided [11]. This care can be provided at different levels. Essential Emergency and Critical Care (EECC) is the first level of care provision and is defined as the effective, low-cost, feasible care that should be provided to all critically ill patients in all parts of all hospitals in the world [11]. The fundamental and universal nature of EECC, when evaluated in light of the current deficiencies in critical care provision and the expenses associated with advanced critical care, indicate that it deserves prioritized investment in global health systems [12, 13]. This approach is particularly relevant for countries like Tanzania, where resources are limited and the need for critical care high [14]. Advanced critical care, including mechanical ventilation and advanced organ support, is more complex and resource-intensive and is typically provided in intensive care units [15]. The resources required for a hospital to be ready to provide EECC have recently been specified in a global consensus study [16] and those for advanced critical care by a group of global experts [12].

Critical care, and in particular EECC, has not been extensively researched in LMICs. The little that is known about availability and quality of care shows large gaps. In Kenya pre-COVID-19, only 58% of beds were in hospitals with an oxygen supply [17]. In Malawi, 90% of hypoxic patients do not receive oxygen [18]. Similarly, studies done in Tanzania [14] and Sierra Leone [19] showed deficiencies in emergency and critical care, particularly in infrastructure, routines and training. With the COVID-19 pandemic and other emerging infections, and the increase in non-communicable diseases, aging populations, militarized conflicts and natural disasters, there has been a realization of the need for increased evidence around critical care and greater research in the field [20–25].

Studies of hospital resources have looked at the reported availability of service-specific resources at facility level [14, 26, 27] including national Service Availability and Readiness Assessments (SARA) and Service Provision Assessments (SPA) [28, 29]. However, such hospital-level availability of resources is not enough to determine if the resources can be utilized to offer care to all those in the wards when in need. Critically ill patients can be in all parts of the hospital and critical care is, by its nature, time-sensitive. A resource that is *available* at hospital-level may not be *ready* beside the bed in the wards for rapid use, and delays in locating and using resources could have life-threatening consequences for the critically ill. Such readiness, while more complicated to assess, is likely to be of greater importance for patient outcomes than hospital availability.

We do not know how ready hospitals are for the provision of critical care. This study aimed to assess the readiness of hospitals in Tanzania for the provision of EECC and the hospital availability of resources for EECC and advanced critical care.

## Methods

### Study design

We conducted an in-depth, cross-sectional study of the availability and readiness of resources for EECC and advanced critical care in all wards in five hospitals in Tanzania.

### Study setting

The study took place in Tanzania, a lower-middle income country in East Africa with a population of 61 million [30]. Tanzania has a pyramidal health system with community dispensaries forming the base, followed by health centres, district hospitals, regional hospitals and finally tertiary hospitals at the apex.

### Study sites

The study included hospitals in Dar-es Salaam and Pwani regions located in eastern Tanzania. A purposive sampling strategy was used to select five hospitals including all levels from tertiary, regional and district hospitals. These hospitals were selected to represent both public and private ownership and different geographical settings including rural and urban settings where the research group had pre-existing relationships. One regional hospital and one district hospital were selected in each region, and one tertiary facility receiving referral patients from all over the country was included. The district-level hospitals function as referral facilities within the district, and usually have five to seven wards including male and female surgical/medical wards, obstetric/gynaecological ward, labour ward, and a paediatric ward. Regional hospitals receive referrals from district hospitals and usually have seven to nine or more wards, including male and female surgical/medical, paediatric, labour, post-natal, ante-natal, obstetrics & gynaecology wards, and an Intensive Care Unit (ICU). Tertiary hospitals, the top referral institutions at the national level are equipped with specialized wards dedicated to medical, surgical, obstetrics & gynaecology, paediatrics, renal, cardiac, High Dependency Units (HDUs), and ICUs.

### Definitions

In this study, we defined “*hospital*-*availability’’* on a hospital level– a resource that is present in the hospital. We define *“ward-readiness”* on a patient level– a resource that is available, is functioning, and is present in the right place, in sufficient amounts, at the right time for its use in the care of patients. Resource requirement lists for EECC (66 items– Supplementary Table 1, Additional File 1) and advanced critical care (161 items–Supplementary Table 2, Additional File 1) developed in previous studies were used for defining hospital-availability and ward-readiness [12, 16]. The lists group resources in the eight categories of infrastructure, human resources, equipment, consumables, drugs, training, guidelines, routines with an additional ninth category of support systems for advanced critical care, (care beyond the critical care location such as laboratory tests and imaging). To align with the previously established EECC requirement lists (Additional File 1) [16], for human resources, a specialist, medical officer, assistant medical officer and a clinical officer were defined as ‘senior health workers’ while nurses of all cadres were defined as ‘health workers’. ‘Routines’ were defined as the systems and processes used in the hospital for managing critically ill patients and ‘guidelines’ defined as written materials–wall charts or booklets– that describe how critically ill patients should be identified or managed.

### Data collection

All the wards and units in the hospitals were visited– outpatient/emergency departments, in-patient wards, high-dependency units (HDUs) and intensive care units (ICUs). All 26 wards in all the district and regional hospitals were visited. The tertiary facility had two identical wards for each department and due to logistical constraints, one of the wards was visited in each of the departments– a total of 11 wards. Data on all the 66 EECC resources and the 161 advanced critical care resources were collected using a structured paper-based questionnaire which consisted of all the categories of the resources. The questionnaire consisted of closed-ended questions with predefined response options (available seen/ available not seen/ not available, quantity and location). The hospital administration was contacted prior to arrival and plans were made on the staff in charges who would accompany the researcher in each ward to facilitate data collection. One researcher, KK, an anaesthesiologist and critical care physician, collected the data accompanied by the staff in charge of each ward who provided contextual information. The researcher utilized the predefined criteria on availability and readiness to manually enter all the data in the tool. Information on the availability, quantities and location of the resources in each ward was collected. For a resource to be considered available, it had to be in the hospital. For a resource to be considered ready, it had to be seen in the ward by the researcher, counted, and information gathered from health workers regarding its consistent availability at the right time when needed for patient care. Additionally, the researcher tested equipment for functionality.

For the purpose of this study, nurses of any cadre were categorized as ‘health worker with ability to identify or to provide care to critically ill patients’. Data on the number of nurses available in each shift were provided by the nurse-in-charge on each ward. A ‘health worker trained in the care of critical illness’ was defined as a health worker who had undergone training including critical care within the last 5 years and data on this was provided by the nurse-in-charge on each ward. Health worker: patient ratios were then calculated using the number of patients in the wards they served. Information on the presence of ‘routines’ was collected from the nurse-in-charge on each ward.

### Data analysis

The data were entered into Microsoft Word and Excel and analyzed using Stata Version 14. For the purpose of determining readiness of equipment and human resources in this study, it was assumed that a ratio of 1 equipment per 20 beds would be adequate for EECC provision, as only a minority of patients are critically ill and equipment is not needed for all. For human resources, one health care worker for every eight patients was considered adequate for the provision of EECC [31], and night-shift staffing levels were used as it was assumed staffing would be lowest at that point. For advanced critical care, a nurse:patient (health worker: patient) ratio of 1:2, 24-hours a day and a clinician:patient (senior health worker: patient) ratio of 1:4 during the day and 1:12 during the night was considered adequate [32]. In the analysis each item was given equal weight.

### Scores

To describe the data, scores were calculated as follows: Each *item readiness score* was calculated for each hospital as the proportion of the wards visited that had the particular EECC resource ready. *The ward readiness score* was calculated for each ward as the proportion of the EECC resources that were ready in the ward. The *hospital readiness score* was calculated as the proportion of all the required EECC resources that were ready in all the wards of the hospital. A *cumulative readiness score* was calculated as a proportion of the EECC resources that were ready across all the five hospitals.

The *hospital availability score* was calculated as the proportion of resources that were available in the hospital. The *cumulative availability score* was calculated as the proportion of the resources that were available across all the five hospitals. *Category readiness and availability scores were* calculated in the same way for each hospital and for all hospitals for each of the eight resource categories.

The readiness findings are presented in a figure using a traffic light colored system by category and hospital, where 100% readiness is green, 99%-75% yellow, 74%-50% orange and less than 50% red. The resources with the highest the lowest *item resource readiness scores* were identified in each category.

## Results

### Characteristics of the hospitals

The bed capacities of the hospitals were between 62 and 1256 (Table 1). All the hospitals had an outpatient department while only the regional and tertiary hospitals had an emergency department. In the district hospitals, all patients were received in the outpatient department. One of the two regional hospitals and the tertiary hospital had an ICU. A total of 37 wards were visited including all wards of the district and regional hospitals and the selected 11 wards of the tertiary hospital.

**Table 1:** Hospital characteristics

Facility	Level	Number of wards	Bed capacity	Units present in each hospital				
				OPD	ED	ICU	PICU	NICU
1	District	5	70	Yes	No	No	No	No
2	District	5	62	Yes	No	No	No	No
3	Regional Referral	7	189	Yes	Yes	Yes	No	No
4	Regional Referral	9	177	Yes	Yes	No	No	No
5	Tertiary	36	1256	Yes	Yes	Yes	Yes	Yes

OPD Out Patient Department, ED Emergency Department; ICU Intensive Care Unit; PICU Paediatric ICU; NICU Neonatal ICU

### Readiness scores

**Fig. 1:**
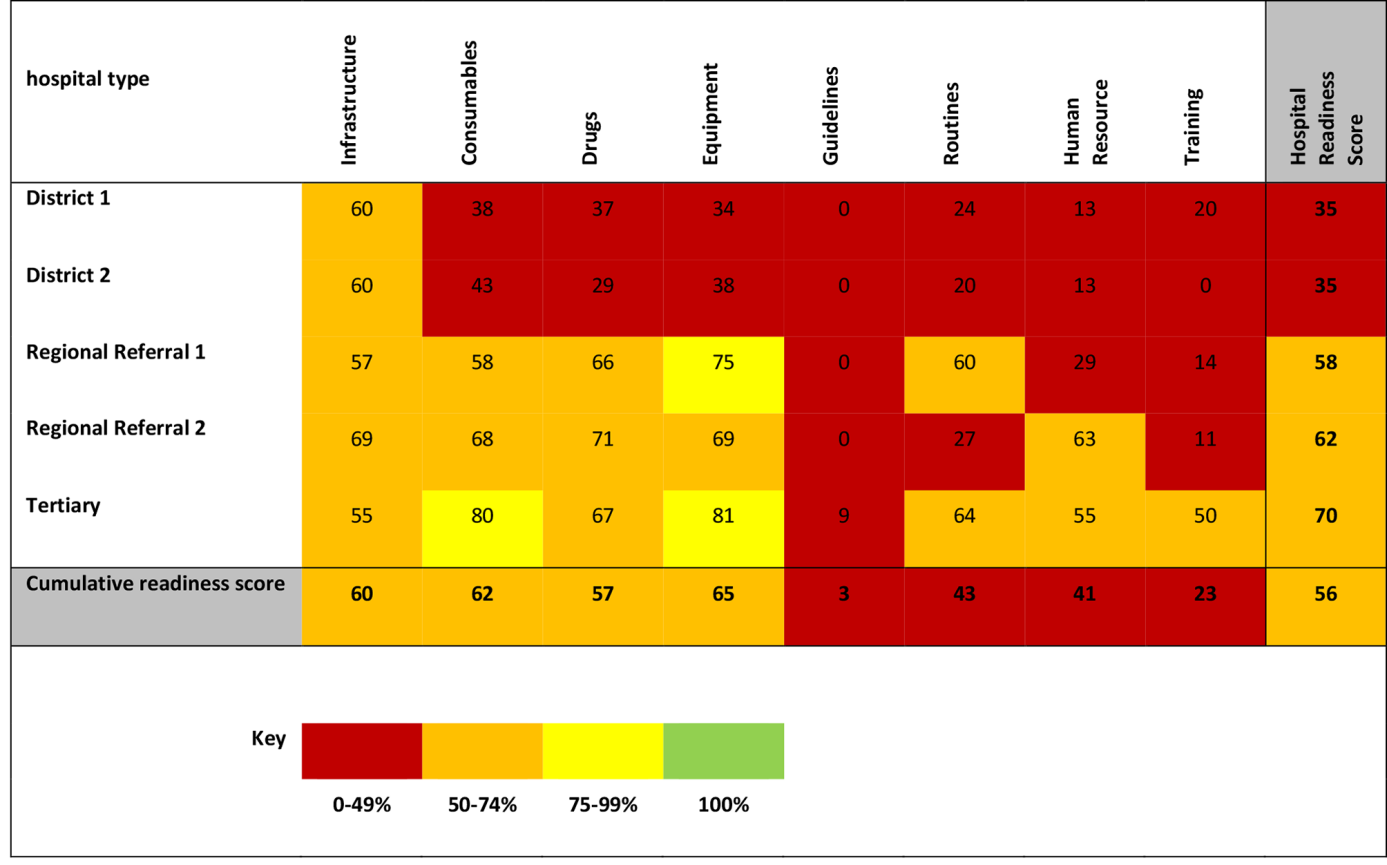
Hospital, category and cumulative readiness scores of EECC

None of the *hospital readiness scores* were 100%. The district hospitals had lower *hospital readiness scores* than the regional and tertiary hospitals with all category readiness scores except infrastructure less than 50%. The *cumulative readiness score* was 56%. None of the categories had a *cumulative category readiness score* of 100%. With the exception of consumables, infrastructure, drugs and equipment, all categories had *cumulative category readiness scores* of less than 50%. Readiness for equipment was the highest (65%) while that of guidelines was the lowest (3%) (Fig. 1).

### Item readiness scores

Only pens had an item readiness score of 100%. The resources with the highest score in each category were: consumables: charts for documentation (97%); routines: a system for critically ill patients to receive care before payment (97%); infrastructure: running water (95%); equipment: blood pressure measuring equipment (87%); drugs: IV fluids (89%); and human resources: sufficient senior health workers (62%).

The resources with the lowest score in each category were: consumables: intraosseous cannulae (3%); training: staff trained to identify and care for critically ill patients (22%); routines: a defined way in which definitive care is given once the patient is stabilized (22%); infrastructure: designated triage area (32%); guidelines: guidelines for identification and care of critically ill patients (3%); equipment: light source (24%); drugs: paracetamol (30%); and human resources: health workers with the ability to identify and care for critically ill patients (30%).

### EECC category readiness scores and category availability scores

**Fig. 2:**
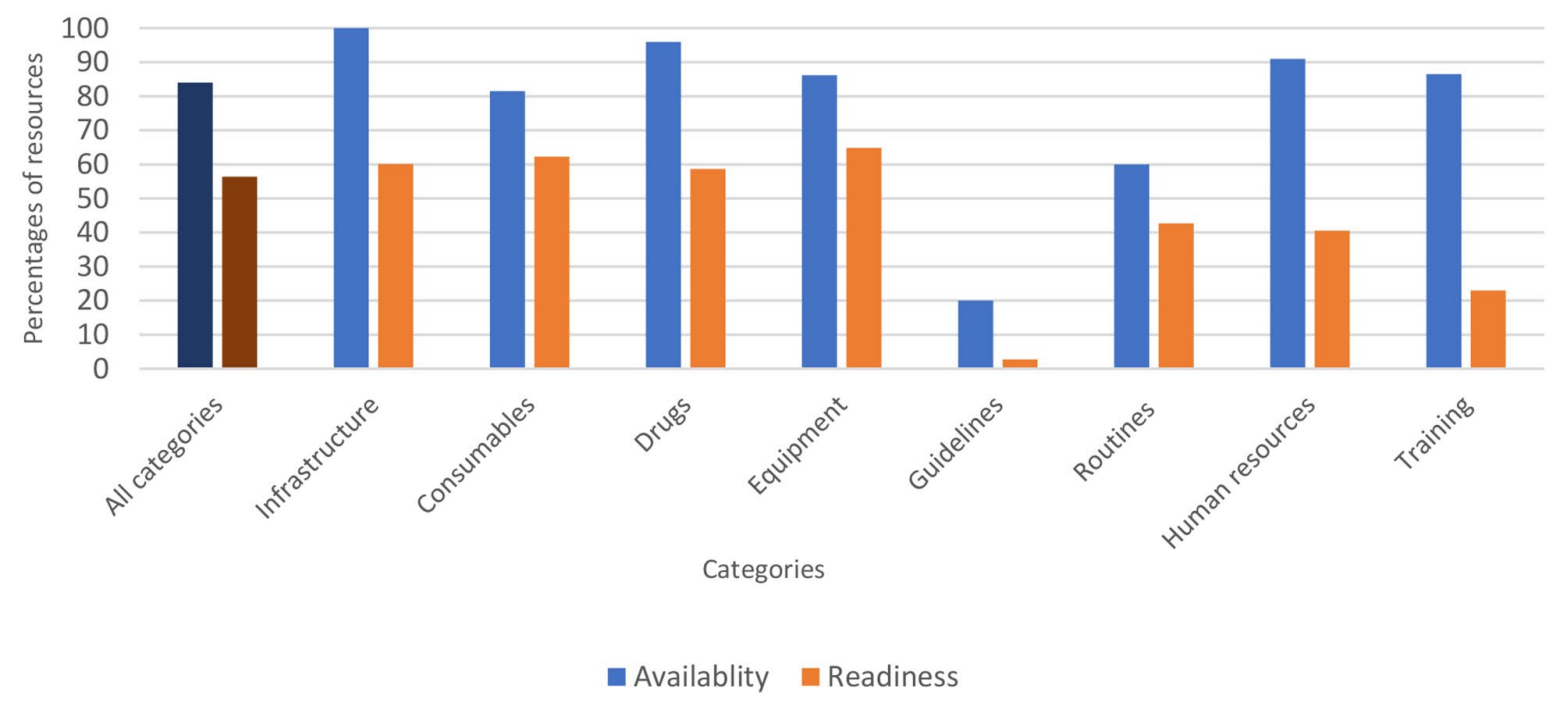
Cumulative category readiness and availability scores for EECC

The *cumulative category availability scores* exceeded 80% for all categories except routines and guidelines. The *cumulative category availability scores* were higher than those of *readiness* for all categories, with notable differences observed between the scores for human resources (91% availability vs. 40% readiness), guidelines (20% availability vs. 3% readiness) and training (86% availability vs. 23% readiness) (Fig. 2).

### Advanced critical care availability scores

The *cumulative availability score* for advanced critical care was 31%. All advanced critical care categories had *cumulative category availability scores* below 50% except for infrastructure (55%) and support systems (78%).

### Comparison between availability scores for EECC and advanced critical care

**Fig. 3:**
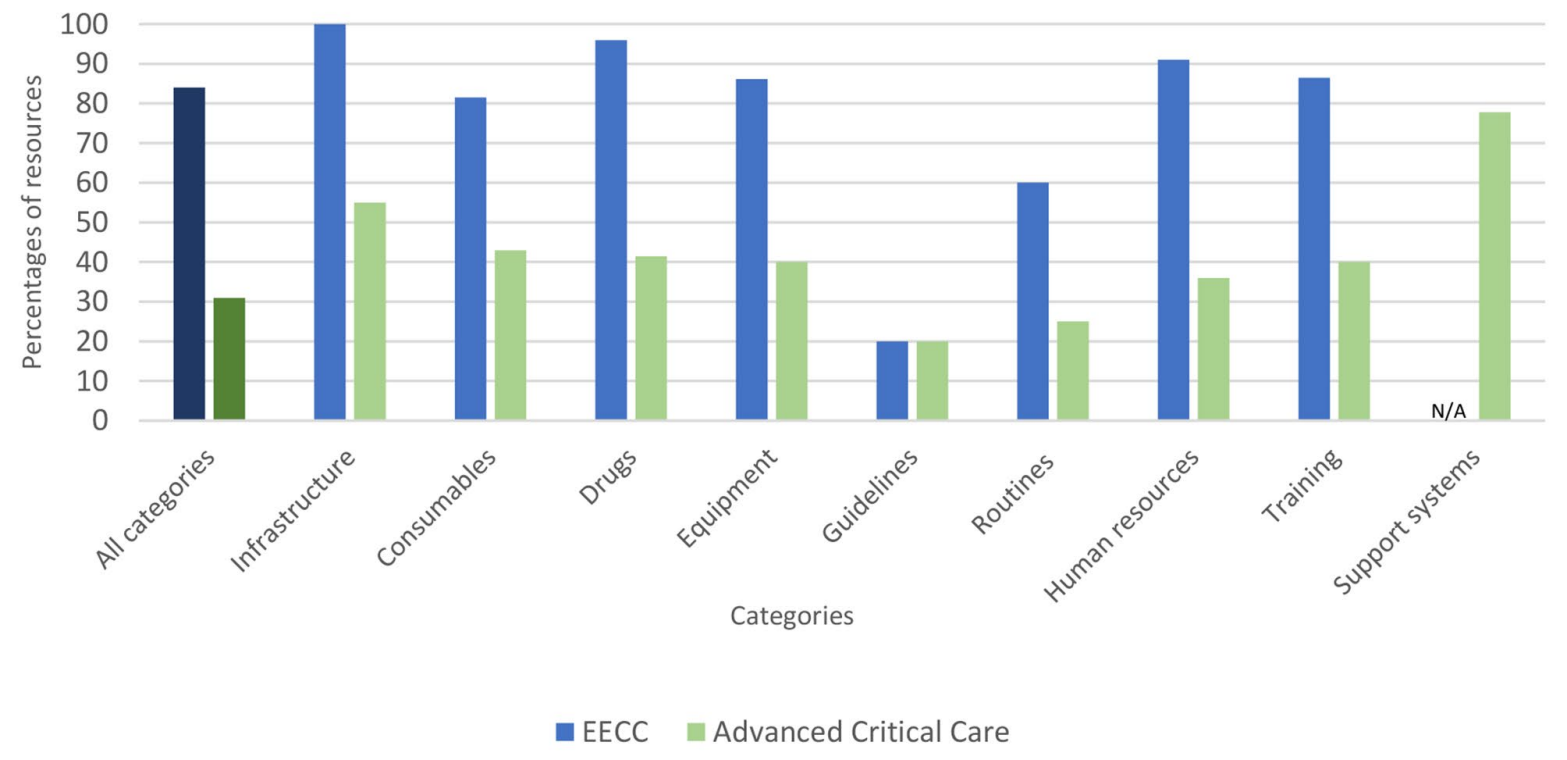
Cumulative category availability scores for EECC and advanced critical care N/A Not Applicable

The *cumulative availability score* for EECC was 84% and the *cumulative availability score* for advanced critical care was 31% (Fig. 3). All *cumulative category availability scores* were higher for EECC than for advanced critical care except for guidelines scores which were low for both.

## Discussion

Hospitals in Tanzania lack readiness for the provision of Essential Emergency and Critical Care. Resources for EECC are usually available somewhere in the hospital– in one ward, a store, a pharmacy or in a locked cupboard– but are often not ready for immediate use in the wards where critically ill patients are cared for. As EECC is defined as the care that all critically ill patients should receive in all hospitals wherever they are located, and has been shown to be of low cost– EECC 17 USD vs. 224 USD for advanced critical care per patient per day [12]– EECC readiness *should* be 100%. Our finding of an EECC readiness of only 56% in five selected hospitals in Tanzania reveals an important barrier to the provision of critical care.

Our results align with research conducted in Tanzania [14], Sierra Leone [15], and Malawi [22] prior to the conceptualization of EECC. These studies uncovered shortcomings in infrastructure, routines, and training for basic emergency and critical care. In our study, the readiness of EECC, particularly in the categories of human resources, training, guidelines, and routines for identifying and treating critically ill patients is low.

Why are hospitals not ready for the provision of such essential care? One possible explanation could be that while the health system is set-up to refer patients from lower-level facilities to higher-level facilities, this is most frequently utilized for complex conditions or for patients requiring specialized or advanced care that is not available in the lower-levels, rather than for critical illness, and furthermore, within hospitals the wards are structured by specialty rather than by severity of illness. Specialty-based wards may prioritize diagnosing and treating underlying conditions, and may focus more on readiness for diagnostic tests, imaging and definitive treatment such as surgery than on life-saving supportive care such as EECC. With the wards lacking readiness for 44% of the resources needed for EECC, there is a large risk of missed chances to deliver feasible, effective life-saving treatment at the bedside. Critically ill patients in these wards are often transferred to better-equipped HDUs or ICUs if there is bed space, which may increase the burden on these units and reduce their capacity to provide care to patients who need more intensive treatment than EECC [33]. Insufficient readiness for EECC in the wards, could result in a substantial number of potentially avertable deaths.

The district hospitals in our study had lower readiness for the provision of EECC compared to the regional and tertiary hospitals. In South Africa, district hospitals were also seen to have low levels of emergency resources for maternal and neonatal care [34]. Lack of adequate resources in lower level facilities, where most patients are cared for, leads to unnecessary referrals to higher facilities - in systems where referrals are also inadequately structured [35]. Higher level facilities may not be adequately resourced for so many patients and can become overburdened and perform ineffectively [36] leading to preventable deaths. Increasing EECC readiness in lower level facilities could be a feasible solution to this problem.

While hospital-level availability of resources for EECC was 84%, that of advanced critical care was lower– 31%. A lack of capacity for advanced care has also been shown in previous studies [15, 37]. Scaling up advanced critical care is costly [38] and complex and might not be effective [39] or cost-effective in the short term for countries with scarce resources such as Tanzania [40]. A study done in Tanzania and Kenya during the COVID-19 pandemic, showed the cost of advanced critical care to be 7 to 9 times higher than that of EECC [12]. Focusing on improving readiness for EECC before scaling up advanced critical care is likely to yield greater benefits and reduce the need for advanced critical care, thus allowing it to be available for the patients where EECC is not enough.

Previous studies have looked only at hospital availability of resources for critical care provision [14, 26–28, 41]. National surveys like SPA and SARA assess service specific availability of resources at hospital-level [26]. By design, surveys assessing hospital availability will overestimate ward readiness. Our findings reveal that this overestimation is substantial and that a high availability of resources at hospital level does not necessarily translate to ward readiness for patient care.

A strength of our study is that our method went beyond commonly used surveys of hospital availability of resources, by making in-depth observations of ward readiness of resources for immediate use for critically ill patients. This provides a clearer picture of the distribution of the available resources within hospitals and their readiness when urgently needed. A limitation of our study is that we were only able to include five hospitals. However, from our experience, we do not expect that there would be substantial differences in other hospitals in the country as systems of care are similar and they are under the same governing system. By design, our study looked at point-prevalence of availability and readiness at the time of the study. It is likely that there might be variations of readiness and availability in different time periods. However, critical illness can occur at any time and place, and readiness for EECC should be 100% at all times in all wards of a hospital.

## Conclusion

Hospitals in Tanzania lack readiness for the provision of essential emergency and critical care– the low-cost, life-saving care for critically ill patients. The resources for EECC were available in hospitals, but they were not ready for the immediate needs of critically ill patients in the wards. In order to provide effective EECC to all patients, improvements are needed around the essential, low-cost resources in hospital wards that are essential for decreasing preventable deaths.

### Electronic supplementary material

Below is the link to the electronic supplementary material.

**Additional File 1: Supplementary Table 1:** The hospital readiness requirements for Essential Emergency and Critical Care. **Supplementary Table 2:** Advanced Critical Care Hospital Readiness Requirements

## Data Availability

Data is available from the corresponding author upon reasonable request.

## Abbreviations

EECC: Essential Emergency and Critical Care
HDU: High Dependency Unit
ICU: Intensive Care Unit
LMICs: Low- and Middle-Income Countries
SARA: Service Availability and Readiness Assessment
SPA: Service Provision Assessment
