# Supplementary material for: Hospital readiness for the provision of care to critically ill patients in Tanzania– an in-depth cross-sectional study
Source: BMC Health Serv Res. 2024 Feb 8;24:182. doi: 10.1186/s12913-024-10616-w (PMC10854052; doi:10.1186/s12913-024-10616-w)
Supplement: Supplementary file 1 — Additional File 1: Supplementary Table 1: The hospital readiness requirements for Essential Emergency and Critical Care. Supplementary Table 2: Advanced Critical Care Hospital Readiness Requirements [file 12913_2024_10616_MOESM1_ESM.docx]

**Additional File 1**

**Supplementary Table 1: The hospital readiness requirements for Essential Emergency and Critical Care(1)**

| **Identification of critical illness** *The following items are required for a hospital to be ready for the identification of critically ill patients* | |
| --- | --- |
| **Category** | **Item** |
| **1.1. Equipment** | 1.1.1 Clock with second-hand  1.1.2 Pulse oximeter and probe  1.1.3 Blood pressure measuring equipment (e.g., sphygmomanometer with a stethoscope)  1.1.4 Blood pressure cuffs of different paediatric and adult sizes  1.1.5 Light source (lamp or flashlight)  1.1.6 Thermometer |
| **1.2 Consumables** | 1.2.1 Soap or hand disinfectant  1.2.2 Examination gloves |
| **1.3 Drugs** | None |
| **1.4 Human resources** | 1.4.1 Health workers with the ability to identify critical illness 24 hours/day |
| **1.5 Training** | 1.5.1 The health workers are trained in the identification of critical illness |
| **1.6 Routines** | 1.6.1 Routines for the identification of critical illness |
| **1.7 Guidelines** | 1.7.1 Guidelines for the identification of critical illness |
| **1.8 Infrastructure** | 1.8.1 Designated triage area (area for the identification of critical illness) in the Out-Patient Department or Emergency Unit (area of the hospital where patients arrive)  1.8.2 Running water |
| **Care of critical illness** *The following items are required for a hospital to be ready to provide the care of critically ill patients* | |
| **2.1 Equipment** | 2.1.1 Suction machine (electric or manual)  2.1.2 Oxygen supply 24 hours/day (cylinder, concentrator (with electricity supply) or piped oxygen)  2.1.3 Flow meter (if using cylinder or piped oxygen)  2.1.4 Leak-free connectors from oxygen source to tubing  2.1.5 Bag valve mask (resuscitator)—neonatal, paediatric and adult sizes  2.1.6 Sharps disposal container  2.1.7 External heat source |
| **2.2 Consumables** | 2.2.1 Suction catheters of paediatric and adult sizes  2.2.2 Guedel airways of paediatric and adult sizes  2.2.3 Pillows  2.2.4 Oxygen tubing  2.2.5 Oxygen nasal prongs  2.2.6 Oxygen face masks of paediatric and adult sizes  2.2.7 Oxygen face masks with reservoir bags of paediatric and adult sizes  2.2.8 Masks for bag valve mask (resuscitator)—neonatal, paediatric and adult sizes  2.2.9 Compression bandages  2.2.10 Plasters or tape  2.2.11 Gauze  2.2.12 Intravenous cannulas of paediatric and adult sizes  2.2.13 Intravenous giving sets  2.2.14 Skin disinfectant for cannulation  2.2.15 Syringes  2.2.16 Nutrition  2.2.17 Nasogastric tubes  2.2.18 Lubricant for nasogastric tube insertion  2.2.19 Intramuscular needles  2.2.20 Intraosseous cannulas of different sizes  2.2.21 Blankets  2.2.22 Facemasks for infection prevention and control  2.2.23 Aprons or gowns  2.2.24 Charts/notes for documentation  2.2.25 Pens |
| **2.3 Drugs** | 2.3.1 Oral rehydration solution  2.3.2 Intravenous crystalloid fluids (eg, normal saline or Ringer’s Lactate)  2.3.3 Intravenous dextrose fluid (eg, 5%, 10% or 50%)  2.3.4 Oxytocin  2.3.5 Epinephrine  2.3.6 Appropriate antibiotics  2.3.7 Diazepam  2.3.8 Magnesium sulphate  2.3.9 Paracetamol  2.3.10 Local anaesthetic (eg, 2% lignocaine) (eg, for intraosseous cannulation) |
| **2.4 Human resources** | 2.4.1 Health workers with the ability to care for critically ill patients 24 hours/day  2.4.2 Senior health worker who can be called to assist with the care of critically ill patients 24 hour/day |
| **2.5 Training** | 2.5.1 The health workers are trained in the care of critically ill patients |
| **2.6 Routines** | 2.6.1 Routines for managing critically ill patients  2.6.2 Routine for the provision of EECC without taking into account patients’ ability to pay  2.6.3 Routines for who and how to call to seek senior help 24 hours/day, 7 days/week  2.6.4 Routines for integrating EECC with other care including the definitive care of the underlying condition (e.g., use of condition-specific guidelines) |
| **2.7 Guidelines** | 2.7.1 Guidelines for the essential care of critically ill patients |
| **2.8 Infrastructure** | 2.8.1 Designated space for the care of critically ill patients (e.g., a bay, ward, high dependency unit)  2.8.2 Areas for separating and managing patients with a suspected or confirmed contagious disease from those without |

**Supplementary Table 2: Advanced Critical Care Hospital Readiness Requirements(2)**

The required resources are divided into categories of equipment, consumables, drugs, human resources, training, routines^[[1]](#footnote-1)^, guidelines^[[2]](#footnote-2)^, infrastructure and support services. Each resource item appears only once, and does not appear if it is included in the EECC HRRL

| **CATEGORY** | **ITEM** |
| --- | --- |
| - 1. EQUIPMENT | 1. Multiparametric patient monitor (ECG, HR, BP, SP02) 2. Flashlight for pupillary reflexes 3. Reflex hammer 4. Scissors 5. Laryngoscope 6. Mackintosh laryngoscope blades of different sizes 7. Straight laryngoscope blades of different sizes 8. Video laryngoscope or fibreoptic flexible laryngoscope 9. Magill’s forceps 10. Cuff manometer 11. Tracheostomy/ percutaneous tracheostomy surgical set 12. Flexible bronchoscope 13. High Flow Oxygen machine 14. High-flow oxygen (piped) 15. Piped Oxygen flow meters 16. CPAP and BiPAP machine 17. Mechanical ventilator 18. Nebuliser for mechanical ventilator 19. Humidifier 20. Air compressor for dilution of oxygen 21. High frequency Oscillatory Ventilator 22. Blood gas analyser 23. Capnograph 24. Minor surgery equipment set 25. Nebuliser 26. Syringe drivers 27. ETT (endo-tracheal tube) holder 28. Invasive blood pressure monitor or module 29. Infusion pumps 30. Glucometers 31. Dialysis machine 32. Defibrillator (monophasic/biphasic) 33. Ultrasound machine with echocardiography capacity and probes 34. Fridge 35. Blood warmer 36. Spine board 37. Pill crusher (for giving enteric medicine) |
| 1. CONSUMABLES | 1. ECG electrodes 2. Rigid neck collars of different sizes 3. Temperature probe for continuous monitoring 4. Urine dipsticks 5. Central venous catheters 6. Arterial catheters 7. Transducer sets for invasive pressure monitoring 8. Central dialysis catheters 9. Nasopharyngeal airways of adult and paediatric sizes 10. Lubricating jelly 11. LMAs (laryngeal mask airways) of adult and paediatric sizes 12. Syringes 20ml and 50ml 13. Endotracheal tubes of adult and paediatric sizes 14. Introducer/stylet for intubation 15. Boogie stylet for intubation 16. Cricothyroidotomy kit 17. Tracheostomy tubes of different sizes 18. High flow nasal catheters 19. CPAP/BiPAP masks 20. HME (Heat & Moisture Exchange) filters 21. Face-straps for CPAP/BIPAP 22. Tubing for ventilator (breathing circuits) 23. Reagents for blood gas analysis 24. Test tubes for blood gas analysis (heparinised) 25. Under water seal drainage system for chest drains 26. Chest drains 27. Sutures 28. Pressure bag able to inflate to 300 mmHg 29. Sterile clothes and drapes 30. Narrow width tape 31. Blood giving set 32. Torniquets for bleeding control 33. Compression stockings 34. Lower-limb pneumatic device 35. Test tubes for sample collection 36. Urinary catheters 37. Urine collection bag with hourly markings 38. Nasogastric tubes 39. Nasogastric feeding bags 40. Bolus-feeding Syringe 41. Glucose strips for glucometer |
| 1. DRUGS | 1. Propofol 2. Ketamine 3. Succinylcholine 4. Atracurium or other non-depolarising muscle relaxant 5. Fentanyl or other rapid-acting opioid 6. Ephedrine or phenylephrine or metaraminol 7. Nitroglycerine 8. Naloxone 9. Neostigmine 10. Atropine 11. Distilled water 12. Salbutamol 13. Ipratroprium 14. Adrenaline 15. Noradrenaline 16. Dobutamine 17. Vasopressin 18. Betablocker e.g. labetolol 19. Amlodipine 20. Nimodipine 21. Heparin 22. Packed red blood cells 23. Platelets 24. Fresh Frozen Plasma 25. Vitamin K 26. Tranexamic acid 27. LMWH e.g. Enoxaparin 28. Mannitol 29. Hypertonic saline 30. Potassium chloride 31. Calcium gluconate 32. Magnesium Phosphate 33. Frusemide 34. Naso-gastric feed 35. Total Parenteral Nutrition feed 36. Insulin 37. Proton Pump Inhibitor eg omeprazole 38. Histamine-2 receptor blocker eg ranitidine 39. Dialysis fluids 40. IV Amiodarone 41. IV Lidocaine 42. Sodium bicarbonate 43. IV paracetamol 44. NSAID e.g. diclofenac 45. Morphine 46. Local anaesthetic agent (block) – e.g. bupivacaine 47. Midazolam 48. Haloperidol 49. Diazepam 50. Clonidine 51. Flumazenil 52. Phenytoin 53. Phenobarbital 54. Levetiracetam 55. Appropriate selection of second line antibiotics (e.g. meropenem, piperacillin, vancomycin) |
| 1. HUMAN RESOURCES | 1. Nursing staff with critical care training or comparable experience in critical care as a nurse: patient ratio 1:2, 24hrs/day, seven days a week 2. Clinicians (e.g. doctors, junior doctors or other health-workers able to diagnose medical conditions, prescribe medications and decide care plans) with Advanced Organ Support training or comparable experience always present in the unit – ratio 1:4 daytime and 1:12 night-time 3. Senior clinician (e.g. doctor with medical degree and experience) with Advanced Organ Support training:    - 1. Daytime: present in the unit for some time every day, on-call for immediate telephone consultation, and always able to be in the unit within 30 minutes      2. Night-time: on-call for immediate telephone consultation and able to be in the unit within 60 minutes 4. Ready access to support from external specialists: (e.g. surgeon, infectious disease specialist/microbiologist, respiratory physician, nephrologist, cardiologist etc) 5. Presence of a rapid response team with an alerting system |
| 1. TRAINING | 1. Training (including simulation) in Advanced Organ Support (e.g. identification of patients requiring AOS, ABCs, medical emergencies, BLS/ALS, crew resource management, teamwork and communication) |
| 1. ROUTINES | 1. Routines for the identification of patients requiring Advanced Organ Support 2. Routines for all of the Advanced Organ Support functions and common problems, emergencies and procedures (e.g. intubation, accidental extubation, cardiac arrest etc) 3. One formal daily multidisciplinary ward round every morning plus an extra shorter ward round every afternoon/evening on weekdays 4. Routines for antibiotic stewardship 5. End-of-life routines |
| 1. GUIDELINES | 1. Guidelines for the identification of patients requiring Advanced Organ Support 2. Guidelines for all the Advanced Organ Support functions (e.g. common problems, emergencies and procedures (e.g. intubation, accidental extubation, cardiac arrest etc) are available and visible to health care workers 3. Guidelines for antibiotic stewardship 4. End-of-life guidelines |
| 1. INFRASTRUCTURE | 1. Intensive Care Unit 2. Adequate space around the patients’ beds 3. Isolation rooms for contagious or immunocompromised patients 4. Rapid communication system |
| 1. SUPPORT SERVICES | 1. Full blood count 2. Urea and Creatinine 3. Electrolytes 4. Lactate 5. Liver function tests 6. Prothrombin Time (INR) and other coagulation tests 7. Blood grouping and cross-matching 8. C-reactive protein 9. Portable chest x-ray (e.g. for endotracheal tube position, nasogastric tube position etc) |

**Total items: 161**

**References**

1. Schell CO, Khalid K, Wharton-Smith A, Oliwa J, Sawe HR, Roy N, et al. Essential Emergency and Critical Care: a consensus among global clinical experts. BMJ Global Health. 2021;6(9):e006585.

2. Guinness L, Kairu A, Kuwawenaruwa A, Khalid K, Awadh K, Were V, et al. Essential emergency and critical care as a health system response to critical illness and the COVID19 pandemic: what does it cost? Cost Effectiveness and Resource Allocation. 2023;21(1):15.

1. “Routines” refers to the system in the hospital for managing critically ill patients. ie. there are routines for the care of critical illness, rather than patients being managed ad hoc. [↑](#footnote-ref-1)
2. “Guidelines” refers to written material (eg. a wall chart or a booklet) that describes how critically ill patients should be identified and managed. [↑](#footnote-ref-2)
